# EPIDEMIOLOGICAL PROFILE OF INJURIES IN PATIENTS WITH HIGH DIAGNOSTIC SUSPICION OF ABUSE

**DOI:** 10.1590/1413-785220253304e290194

**Published:** 2025-09-08

**Authors:** Isaias Silva Ribeiro de Souza, Victor Peyneau Poncio, Matheus Giovanni Medina Molina, Georges Akira Shigekiyo Perera, Luiz Fernando Cocco, Eiffel Tsuyoshi Dobashi

**Affiliations:** 1 Universidade Federal de Sao Paulo, Escola Paulista de Medicina, Departamento de Ortopedia e Traumatologia, Sao Paulo, SP, Brazil.

**Keywords:** Child Abuse, Child Maltreatment, Epidemiology, Aggression, Fractures, Bone, Child, Abuso de Crianças, Maus-Tratos de Menores, Epidemiologia, Agressão, Fraturas Ósseas, Criança

## Abstract

**Objective:**

This study aimed to analyze the epidemiological profile of child abuse cases treated at Hospital Geral de Pirajussara, São Paulo, and to understand the characteristics of associated injuries.

**Methods:**

A retrospective cross-sectional study was conducted by reviewing medical records of patients suspected of abuse, aged 18 years or younger, from January 2012 to December 2022. Data on sex, age, trauma mechanism, presence of fractures, and outcomes were analyzed.

**Results:**

A total of 58 records were included. Most cases involved adolescents (50%, n=29). The most common abuse mechanism was physical force (36.21%, n=21), followed by direct trauma by object (13.79%, n=8). Fractures were present in 41.38% of cases (n=24), with skull and facial fractures being the most frequent (33.33%, n=7). Brain injuries were the most common associated injuries (42.42%, n=14). Most cases (77.59%, n=45) were discharged with an average hospital stay of 9 days.

**Conclusions:**

Abuse is prevalent among young children under 1 year and adolescents (13-18 years). Identified patterns of injuries and abuse mechanisms highlight the need for stringent screening and management protocols. Continuous training and vigilance are crucial for effective prevention and intervention. ***Level of Evidence III; Cross-Sectional Retrospective Study.***

## RESUMO

**Objetivo:**

Analisar o perfil epidemiológico de crianças vítimas de maus-tratos atendidas no Hospital Geral de Pirajussara, São Paulo, e investigar as características das lesões associadas.

**Métodos:**

Conduzido um estudo transversal retrospectivo com revisão de prontuários dos pacientes com suspeita de maus-tratos, idade igual ou inferior a 18 anos, entre janeiro de 2012 e dezembro de 2022. Analisaram-se dados sobre sexo, idade, mecanismo de trauma, presença de fraturas e desfecho.

**Resultados:**

Dos 58 prontuários analisados, 50% (n=29) referiam-se a adolescentes. O abuso mais comum foi por força corporal (36,21%, n=21), seguido por trauma direto por objeto (13,79%, n=8). Fraturas foram encontradas em 41,38% dos casos (n=24), com maior frequência em crânio e face (33,33%, n=7). Lesões cerebrais foram mais prevalentes entre as lesões associadas (42,42%, n=14). A maioria dos pacientes (77,59%, n=45) recebeu alta apresentando internação média de 9 dias.

**Conclusões:**

Maus-tratos são prevalentes em crianças menores de 1 ano e adolescentes (13-18 anos). Os padrões de lesões e mecanismos de abuso evidenciam a necessidade de protocolos rigorosos para triagem e manejo. A capacitação contínua e a vigilância são essenciais para a prevenção e intervenção eficaz. ***Nível de Evidência: III; Estudo Transversal Retrospectivo.***

## INTRODUCTION

Child abuse and ill-treatment are defined by the Child Abuse Prevention and Treatment Act (the Child Abuse Prevention and Treatment Act, CAPTA) as emotional or physical harm, sexual abuse, exploitation and imminent risk of death inflicted on persons under the age of 18.^1^ Nationally, Articles 4 and 5 of the Child and Adolescent Statute of 13 July 1990 define that every child should be assured by the public authority of the rights to life, leisure, respect, food and family and community coexistence without physical or mental discrimination, without violence, without cruelty, without oppression and without exploitation.^2^ However, it is presumable that there is a subnotification of many of the cases, especially in our environment where, countless incidents are not diagnosed. It is considered as the only criterion of confirmation of the fact, especially in child abuse, the confession of the abuser. The most commonly affected age group is children under 2 years of age, however, in children under 1 year of age, the incidence corresponds to 50% of all abuse injuries.^3^

As for beaten child syndrome, a condition in which the child is a victim of non-accidental physical trauma, by one or more of its carers, its incidence is 1 in 100 children. Among those affected, about 2 to 3% progress to death. In Toronto's Hospital for Sick Children, the incidence observed in this service was three times higher than in other conditions such as congenital impotence.^4^

The repercussions on the life of a child victim of ill-treatment are countless. When exposed to such abuse, they may develop mental health problems throughout their development. Among them we can cite anxiety, depression, sleep disorders, post-traumatic stress disorders, lack of concentration in school and hypervigilance.^5^ In addition, the population when it reaches adulthood is most seen in mental health services and is more prone to self-harassment and suicide.^6^

The most commonly found lesions are those of soft tissues and fractures that are present in up to 20% of patients. Given this fact, for the assessment of a possible case of ill-treatment, orthopedists will often be required for the assessment of this population.^7^ An important fact to consider is that a single fracture cannot be considered, in isolation, to define a framework of ill-treatment. The entire clinical and social context of the patient and the family involved should be considered.

However, there are patterns of fractures that should increase suspicion and generate a warning for a possible ill-treatment injury, which are: scapula, sternum, metalizarias, posterior coastal arches and thinning processes.^8^ It is important to emphasize that even when we have fractures of low specificity, but with a confusing and/or unconnected history, this injury becomes highly suspicious for ill-treatment.

In addition, in the suspicion of child abuse, we should consider some differential diagnoses such as rachitis or imperfect osteogenesis that may present similar radiographic signs. Considering the fractures of the ribs, in bone fragility, these occur more frequently in its lateral portion, however in the normal skeleton the involvement is postromedial which increases the suspicion of a non-accidental trauma.^9^ Therefore, it is of extreme importance that requests for radiographies should be standardized for determination of cases follow strict protocols to perform the screening of possible other fractures at the same time or in previous periods. The fractures that have moderate specificity are the epiphyse separations, fractures of the phalanges and vertebral bodies. The fractures of low specificity, or more likely to be accidental, are the diaphysic fractures of long bones (at less than 1 year old), clavicular fractures and linear fractures of the skull.^10^ Due to the difficult diagnosis and high clinical repercussions in the cases, more factors should be established to strengthen suspected fractures by ill-treatment, in order to avoid possible adverse outcomes for patients.

Therefore, this work was developed to establish the epidemiological profile of patients victims of ill-treatment in a reference hospital in São Paulo. A better understanding of injuries in this population may be useful in developing protocols for care and protection of the children involved. The conduct of this research was motivated by the scarcity of articles from the national orthopedic literature.

## MATERIAL AND METHODS

The study was approved by the Research Ethics Committee (CEP) of the General Hospital of Pirajussara with the Certificate of Presentation of Ethical Appreciation number 74005723.8.0000.5450 issued by the Institute of Gastroenterology of São Paulo - IGESP, through the Brazil platform. The procedures followed the standards of the aforementioned Ethical Committee on Human Experiences of the Federal University of São Paulo and are in accordance with the 1995 Helsinki Declaration.

### Design of the study

A cross-sectional retrospective study was conducted based on the descriptive review of data collected from electronic records of patients suspected of ill-treatment aged 18 or less treated at the General Hospital of Pirajussara, which comprises the municipalities of Embu das Artes and Taboão da Serra – SP, from January 2012 to December 2022.

With respect to the Terms of Free and Informed Consent (TCLE), a waiver and exemption from the application of this tool was requested, as this study used only secondary data obtained from the study of material already collected for assistance purposes and the review of medical records with information concerning patients.

### Population

The population composition was obtained by selecting all the cases notified to the Epidemiological Surveillance by the medical or social care team, considering the suspected or confirmed cases of ill-treatment treated at the General Hospital of Pirajussara.

We included all patients followed between January 2012 and December 2022, of both sexes, aged under 18, with positive notification from the Protection Council for Abuse and signature of the Free Informed Consent Terms that contained data in electronic medical records. The cases that did not meet the inclusion criteria were not selected for sampling.

### Procedures

Data were collected regarding gender, age, origin, mechanism of trauma, presence of fracture, laterality (where applicable), presence of associated injuries, time of hospitalization and outcome. An epidemiological analysis of the data obtained was carried out with the aim of establishing the profile of the studied sample and comparing it with available literature data on the subject.

### Gathering and obtaining data

The data was obtained through the review of electronic logs and the information relevant to the database composition was tabulated in Microsoft Excel® 2010 Software spreadsheet (Microsoft Corporation®, San Diego, USA).

### Statistical analysis

The data obtained was analyzed using Microsoft Excel® 2010 Software (Microsoft Corporation®, San Diego, USA). The categorial data were presented in the form of frequency and percentage and the continuous numerical data in the form of sample average and standard deviation. The analyses were carried out to characterize the sample and establish an epidemiological profile.

## RESULTS

Having met the eligibility criteria for sample composition, 58 records were included for analysis. Our study included patients from the municipalities of Taboão da Serra (41.38%), Embu das Artes (36.21%) and São Paulo (10.34%). Table 1 shows the other municipalities considered by our sample.

**Table 1:** Characterization of the sample according to municipalities of origin of patients (N=58).

	N (%)
Barueri	1 (1.72)
Carapicuiba	1 (1.72)
Cotia	1 (1.72)
Embu of the Arts	21 (36.21)
Ibiuna	1 (1.72)
Itaberada	1 (1.72)
Itapecerica da Serra	1 (1.72)
Itapevi	1 (1.72)
São Paulo	6 (10.34)
Taboão da Serra	24 (41.38)

Of the total of our sample, 31 (53.45%) were male and 27 (46.55%) female, whose average age was 10.2 years (1 day as minimum age and 18 years as maximum age); lower for boys (9.72 × 10.75 and 1 day X 1 month, respectively) and maximum age of 18 years for both sexes. The age distribution is concentrated among younger children, from early childhood, 18 present age from 0 to 6 years (20.69%) and adolescence, 29 present age between 13 to 18 years (50%). The cases studied occurred mainly from 2015 to 2018, with 18 cases occurring in 2015 (31.03%), in 2016 were 12 cases (20.69%), in 2017 with 7 cases (12.07%) and 2018 also with 7 cases (12.07%), with the least occurrence from 2019. In the years when there was a higher incidence of cases of ill-treatment, the prevalence was higher in male individuals and in the 4 years of lower incidence, there was a higher prevalence in female. (Table 2)

**Table 2:** Characterization of the sample by age and cases per year according to sex (N=58).

		Total	Male	Female
N (%)		58	31 (53.45)	27 (46.55)
Average age (years) (SD)		10.20 (6.55)	9.72 (6.8)	10.75 (6.33)
Min-Max		1 day - 18 years	1 day - 18 years	1 month - 18 years
Division by age group N(%)	< 1 year	12 (20.69)	7 (12.07)	5 (8.62)
	1-6 years	6 (10.34)	4 (6.9)	2 (3.44)
	7-12 years	11 (18.96)	7 (12.07)	4 (6.89)
	13-18 years	29 (50)	13 (22.41)	16 (27.59)
Cases per year N(%)	2015	18 (31.03)	14 (24.14)	4 (6.9)
	2016	12 (20.69)	4 (6.9)	8 (13.79)
	2017	7 (12.07)	4 (6.9)	3 (5.17)
	2018	7 (12.07)	4 (6.9)	3 (5.17)
	2019	4 (6.9)	1 (1.72)	3 (5.17)
	2020	4 (6.9)	1 (1.72)	3 (5.17)
	2021	4 (6.9)	2 (3.45)	2 (3.44)
	2022	2 (3.45)	1 (1.72)	1 (1.72)

According to Table 3, the most frequent mechanism of abuse was physical force, accounting for 20 cases (34.49%). The minimum age was 1 month, the maximum age was 18 years, and the average age was 10.51 years; however, the ages were widely dispersed (SD = 6.55). The distribution of boys and girls was similar (N = 10 and 11, respectively). Direct traumas per object occupied the second place in prevalence with 8 occurrences (13.79%) and beating, the third with 6 cases (10.34%), and this was most common in older children (average age 15.17 years, SD = 2.32). The lowest age was observed for the abandonment mechanism (1 day and 2 months) and the highest for white gun injury (average 15.5 years). Drowning and bite were the mechanisms of lowest prevalence, with only 1 case. Sexual abuse was observed in 2 cases (3.45%), only in female patients, aged 14 and 16.

**Table 3:** Age, gender and time of admission according to abuse mechanism, fracture, associated injuries and outcome (N=58).

					Male		Female		Internation		
		N (%)	Min-max	Middle Age (SD)	N (%)	Age Mean (SD)	N (%)	Middle Age (SD)	Yes N(%)	No N(%)	Duration (days) Mean (SD)
	
Mechanism of abuse	Abandonment of incapable	2 (3.45)	1 day - 2 months	0.09 (0.12)	2 (3.45)	0.09 (0.12)	0		2 (3.45)	0	3.5 (3.54)
	Sexual abuse	2 (3.45)	14 - 16 years	15 (1.41)	0		2 (3.45)	15 (1.41)	2 (3.45)	0	2.5 (2.12)
	Affecting	1 (1.72)	9 years	9	1 (1.72)	9	0		1 (1.72)	0	7
	Spaning	6 (10.34)	12 - 18 years	15.17 (2.32)	4 (6.9)	14.5 (2.65)	2 (3.45)	16.5 (0.71)	5 (8.62)	1 (1.72)	33.8 (39.19)
	White gun injury	4 (6.9)	13 - 18 years	15.5 (3.54)	1 (1.72)	18	3 (5.17)	16.33 (2.89)	4 (6.9)	0	5 (2.94)
	Fire gun injury	4 (6.9)	5 - 18 years	11.5 (6.95)	3 (5.17)	13.33 (7.23)	1 (1.72)	6	2 (3.45)	2 (3.45)	4 (4.24)
	Body strength	21 (36.21)	1 month - 18 years	10.51 (6.55)	10 (17.24)	9.85 (7.32)	11 (18.97)	11.10 (6.07)	15 (25.86)	6 (10.34)	4.13 (3.44)
	Human bite	1 (1.72)	15 years	15	0		1 (1.72)	15	0	1 (1.72)	
	Negligence	2 (3.45)	5 months - 11 years	5.71 (7.48)	1 (1.72)	11	1 (1.72)	0.42	2 (3.45)	0	6 (0)
	Direct Trauma by Object	8 (13.79)	3 - 18 years	10.88 (5.54)	5 (8.62)	9.8 (6.83)	3 (5.17)	12.67 (2.52)	5 (8.62)	3 (5.17)	4 (3.67)
	Fall	5 (8.62)	1 month - 12 years	3.6 (5.12)	3 (5.17)	5.78 (5.87)	2 (3.45)	0.33 (0.35)	5 (8.62)	0	17.8 (19.77)
	No data	3 (5.17)	2 months - 14 years	4.78 (7.98)	1 (1.72)	0.17	2 (3.45)	7.09 (9.78)	1 (1.72)	2 (3.45)	3
Fraud	Yes*	21 (36.21)	1 month - 18 years	9.66 (7.13)	14 (24.14)	9.49 (7.48)	7 (12.07)	10.01 (6.91)	20 (34.48)	1 (1.72)	9.45 (19.54)
	No	34 (58.62)	1 day - 18 years	10.66 (6.28)	15 (25.86)	10.34 (6.28)	19 (32.76)	10.91 (6.44)	21 (36.21)	13 (22.41)	7.14 (10.50)
	No data	3 (5.17)	2 months - 13 years	8.72 (7.41)	2 (3.45)	6.59 (9.07)	1 (1.72)	13	2 (3.45)	1 (1.72)	30.5 (41.72)
*Yes	Hand	4 (19.05)	15 - 17 years	15.5 (1)	3 (14.29)	15.67 (1.15)	1 (4.76)	15	3 (5.17)	1 (1.72)	3 (1)
	Lower members	5 (23.81)	1 month - 17 years	4.85 (7.08)	4 (19.05)	5.56 (7.96)	1 (4.76)	2	5 (8.62)	0	6 (5.15)
	Superior members	5 (23.81)	1 month - 15 years	7.82 (6.59)	1 (4.76)	3	4 (19.05)	9.02 (6.94)	5 (8.62)	0	3.6 (1.67)
	Crane and face	7 (33.33)	4 months - 18 years	9.8 (8.25)	6 (28.57)	8.6 (8.34)	1 (4.76)	17	7 (12.07)	0	16.57 (32.42)
Associated injuries	Yes**	33 (56.9)	1 day - 18 years	9.49 (7.15)	16 (27.59)	8.86 (7.03)	17 (29.31)	10.08 (7.42)	27 (46.55)	6 (10.34)	7.07 (9.72)
	No	23 (39.66)	1 month - 18 years	11.53 (5.42)	13 (22.41)	11.25 (6.47)	10 (17.24)	11.9 (3.96)	15 (25.86)	8 (13.79)	9.93 (22.4)
	No data	2 (3.45)	2 months - 13 years	6.59 (9.07)	2 (3.45)	6.59 (9.07)	0		1 (1.72)	1 (1.72)	60
**Yes	Brain	14 (42.42)	1 day - 18 years	6.28 (7.33)	8 (24.24)	6.57 (7.1)	6 (18.18)	5.89 (8.3)	8 (13.79)	6 (10.34)	6 (6.26)
	Torax	5 (15.15)	2 months - 18 years	10.83 (7.99)	4 (12.12)	10.29 (9.12)	1 (3.03)	13	5 (8.62)	0	7.2 (0.84)
	Abdomen	3 (9.09)	10 - 18 years	14.33 (4.04)	2 (6.06)	14 (5.66)	1 (3.03)	15	3 (5.17)	0	6 (4.58)
	Nerves and tendons	2 (6.06)	14 - 18 years	16 (2.83)	0		2 (6.06)	16 (2.83)	2 (3.45)	0	3 (0)
Outcome	Altab	45 (77.59)	1 month - 18 years	10.64 (6.19)	22 (37.93)	9.94 (6.5)	23 (39.66)	11.31 (5.93)	36 (62.07)	9 (15.52)	9 (16.31)
	Death	6 (10.34)	1 day - 18 years	3.91 (7.17)	4 (6.9)	5.79 (8.46)	2 (3.45)	0.14 (0.04)	1 (1.72)	5 (8.62)	1
	Judicial transfer	1 (1.72)	11 years	11	1 (1.72)	11	0		1 (1.72)	0	8
	Escape	1 (1.72)	4 months	0.33	1 (1.72)	0.33	0		1 (1.72)	0	4
	Ambulatory route	1 (1.72)	18 years	18	1 (1.72)	18	0		1 (1.72)	0	1
	No data	4 (6.9)	13 - 17 years	15 (1.63)	2 (3.45)	15 (2.83)	2 (3.45)	15 (0)	3 (5.17)	1 (1.72)	20.67 (34.06)

The prevalence of hospitalization and non-hospitalization was calculated compared to the general sample (N=58). The prevalence of fractures and associated lesions was calculated compared to the general sample (N=58) and the prevalence of specific fractures and associated lesions was calculated compared to the positive fractural cases sample (N=21) and associated lesions (N=33). ^a^Direct trauma per object includes contundent object (aspirator); perforating-cutting object; direct trauma by skate and direct trauma by unspecified objects. ^a^‘Fall’ includes falls from objects such as a plate, bed, or crib, as well as falls from different floor levels. ^b^Includes post-surgical discharge; post-procedures; post-evaluation of social service; post-evaluation of custodial council and judicial discharge after loss of custody.

* 38.1% (N=8) presented laterality to the right, 33.33% (N=7) to the left and 14.29% (N=3) did not contain data. *Hand fractures include phalanges and metacarpal. *Lower limb fractures include femur and tibia. *Upper limb fractures include humerus and forearm. *Face and skull fractures include skull, occipit, orbit, jaw, and nasal fracture.

** Brain injuries include TCE, extradural hematoma, intracranial hypertension and cerebral death.** Thoracic lesions include pneumothorax and increased mediastinum. ** Abdominal lesions include liver, kidney, splenic and unspecified lesions.

In 34 cases (58.62%) there was no fracture, and among the cases with bone injury, the skull and the bones of the face accounted for one third of the occurrences with 7 cases (33.33%). The youngest was 4 months old, with occipital fractures from falling neck. Upper limb fractures were most observed in girls, 4 cases (19.05%), lower limb fractures in boys, 4 cases (19.05%), and hand fractures in older boys with 3 cases (14.29%) with an average of 15.5 years.

Traumatic head lesions with impaired central nervous system were the most prevalent among the associated lesions, and were observed in 14 patients (42.42%) of the 33 cases. Thorax injuries occupied the second place, representing 15.15% of associated injuries and were mainly related to the use of guns in older patients: of the 5 cases, 2 were for white gun (18 and 13 years old) and 1 for firearm (18 years old).

In our study, 45 (77.59%) of the cases developed with medical or hospital discharge, with an average duration of 9 days of hospitalization, but with large dispersion, ranging from 1 day to 3 months (SD = 16.31). Mechanism by beating and the presence of associated skull and face fractures showed longer hospitalization times (average 33.8 and 16.57 days, respectively).

## DISCUSSION

The main data of this survey are arranged in Tables 1, 2 and 3 which revealed worrying patterns with notable peaks among children under 1 year and adolescents older than 14 years, compromising both sexes. The results indicate that individuals under the age of 1 year often experience severe physical abuse. Such observations have been evidenced by other studies that point to a high incidence of fractures associated with abuse in infants.^1,11^ These fractures are often diagnosed in paediatric emergencies, where collaboration with child protection services is essential to identify and conduct these cases effectively.^7^ The complexity of the signs of abuse in infants are often confused with other medical conditions. We therefore emphasize that an appropriate multidisciplinary approach can ensure an adequate response and the necessary protection for these vulnerable children.

On the other hand, among adolescents over the age of 14, cases of abuse reveal a pattern of violence that may be related to different factors such as psychological pressure and exacerbated family conflicts during adolescence.^5^ Some studies show that there may be more complex forms of abuse in this age group, including psychological abuse and negligence that often result in psychiatric disorders and self-destructive behaviors.^6^ There are research reports that show that mental health interventions and support programs for adolescents are crucial to mitigate the long-term impacts of abuse.^3^ In addition, the implementation of public policies that promote education and awareness about child abuse and its suspicion can help detect and prevent these cases.

The data found in our study demonstrate consistent patterns that corroborate the evidence and recommendations found in the literature on child abuse. According to Weinstein and Flynn^1^ and the review of Kemp et al.,^8^ the prevalence of fractures in child abuse victims is a significant indicator of non-accidental trauma. Our research shows that early childhood had the highest rate of fractures (36.21%), with the highest incidence in the skull and face, which is consistent with the findings that young children are especially vulnerable to severe fractures from physical abuse.^1^

In addition, the traumatic mechanisms identified, such as the application of body force and beating, reflect the most common forms of physical abuse described in the literature.^3,7^ In second childhood and adolescence, the prevalence of beating and white gun injuries, as well as associated injuries, such as cranial trauma, corroborate the observations that adolescents often face more severe and varied abuses.^5,12^ Hospitalization discharge as the predominant outcome, although the most frequent, highlights the need to carry out continuous surveillance, as the literature points to the importance of early interventions and follow-up to minimize long-term complications.^6^

Over the course of early childhood, victims of abuse have a high prevalence of injuries associated with direct physical trauma. Table 3 shows that the average age of the victims of physical strength is 10.51 years, indicating that this form of trauma is significant in younger children. The data also reveal that neglect and abandonment are commonly observed mechanisms, although less frequent, with an average age of around 5.71 years. These findings are consistent with studies that highlight the vulnerability of young children to direct physical abuse and neglect, as indicated by research on child abuse patterns.^3,12^

In second childhood, physical abuse, especially by applying physical force and trauma directly per object, remains very prevalent. Fractures in the lower and upper limbs are common, reflecting the intensity of physical abuse. The average age for limb fractures is approximately 7.82 years for the superior and 4.85 years for the inferior, demonstrating a continuity in the pattern of physical abuse as children grow up. The results found coincide with the findings of studies such as those of Kemp et al.^8^ that show a high incidence of fractures in school-age children.^8,12^ In addition, abuse by excessive body force application and direct trauma per object continue to be prevalent.

In adolescence, patterns of abuse become even more complex and severe. We observed an increase in the severity and diversity of injuries, including beating, gunshot injuries and white gun injuries. The average age considering the beating was 15.17 years, indicating that severe physical abuse tends to occur in older ages. The hospitalization rate for these injuries was significantly high, with an average duration of 33.8 days for the beating. These data reflect the severity of abuses faced by adolescents and are in line with the literature that highlights an increase in the severity of injuries with age.^9,14,15^ The pattern of fractures and associated injuries observed suggests the need for more rigorous approaches to identify and treat cases of abuse in adolescents.

The data compiled in this paper reveals worrying patterns of victimization throughout the different stages of childhood development. The results show that victimization is significant in four distinct age groups: first and second childhood and adolescence, with variations in the type and severity of associated lesions. In early childhood, we observe a high prevalence of severe physical abuse, especially fractures associated with non-accidental trauma. The analysis of the data indicates that the highest rate of bone lesions occurs in this age group, with a prevalence of 36.21% of these in the skull and face. These findings are consistent with the literature describing the vulnerability of young children to severe fractures due to physical abuse.^1,11^

We also note that in early childhood, direct physical abuse and neglect are predominant, while in second childhood, physical abuse persists, with emphasis on fractures. In adolescence, the pattern of abuse includes more serious and varied forms of violence, such as beating and gun injuries. These findings are consistent with the literature, which points to a progression in the severity and complexity of injuries as children age.^4,5^

Our study showed that in 17 of the 58 cases evaluated occurred in patients up to 5 years old, with 12 under 1 year and 5 positive for fracture, strengthening the idea that a series X-ray investigation is considered valid strategies of support for the establishment of proper diagnosis and management. We believe that our results point to the need for special attention to the younger population with emphasis on the investigation of hidden fractures and in cases of suspicion, and protective hospitalization may be of great value.

Due to the morbidity of brain injuries from abuse, neuroimaging as screening for children under investigation of abuse is recommended by the American Academy of Pediatrics (RR). Consistently, our results indicate that four of the six deaths were observed in infants < 1 year (1 day, 41 days, 2 months and 2 months), of which 3 had associated brain injury. All cases of death, except one that presented incomplete data, presented associated brain injury (3 TCE, 1 intracranial hypertension and 1 cerebral death) and associated skull and face fractures showed to be associated with longer hospitalization time (average of 16.57 days).

Furthermore, our study observed, among the positive cases for fracture, a prevalence of 23.81% of fractures of upper and lower limbs and of 19.05% for fractures of the hand. Although skull and face fractures were more prevalent individually, long bone fractures of the appendicular skeleton represented 14 of the 21 cases.

This study demonstrates that there has been a reduction in the incidence of cases of ill-treatment since 2015, with the number of cases stable from 2019 to 2021. In 41.38% of cases of ill-treatment occurred in young people aged 14 to 18 and 66.66% of deaths occurred in children under 1 year. whose results are close to those of the national scene. In Brazil, from 2016 to 2020, of 35,000 violent deaths to 19 years old, more than 31,000 were between 15 and 19 years old. The lethal violence peaked between 2016 and 2017 and fell in subsequent years. However, the number of deaths increases in the 0 to 4 year range (EE). Similarly, in the United States, 80% of child abuse or negligence deaths occur in children under the age of 4 (RR). Although the prospect of reducing the number of cases is favourable, we believe that professional training is necessary in addressing and managing cases of ill-treatment in the younger age group.

Our study presents limitations such as having been developed in a single center and of retrospective collection, which may have limited the acquisition of important data not archived in records, in addition to the collection of text-free file information, which may have made it difficult to standardize the data. We also highlighted the great diversity of the data, with multiple cases isolated from the variables analyzed, which may have been a bias factor for the grouping of the results. Despite the collection of all the cases notified to the Council, the sample limitation, with few cases per year, made longitudinal epidemiological time evaluation difficult. We also mentioned the possible phase-out of expertise in the identification, suspecting and conducting of cases of ill-treatment, due to the usual reduced frequency of such occurrences in hospital daily life. Among the strengths of this study, we highlight its basis in a reference hospital serving approximately 570,187 residents of the municipalities of Taboão da Serra and Embu das Artes. Additionally, the cases were managed by final-year medical residents specializing in orthopedics, which conferred greater technical expertise and specialization in the orthopedic evaluation of child abuse cases. Finally, we cite the public health service as a possible factor of representativity of the results obtained on a large scale and fidelity to the national scene

In summary, the vulnerability of young children to serious fractures and direct physical abuse persists from early childhood to adolescence, although the complexity and severity of the injuries increase with age. The continuation of physical abuse patterns and the emergence of more complex forms of abuse in adolescence highlight the need for a multidisciplinary approach and public policies focused on prevention and early intervention. The literature recommendations, which emphasize the importance of continuous surveillance and education on signs of abuse, are crucial to improving the detection and treatment of these cases, as described by recent studies.^2,3,7^

## CONCLUSION

Child ill-treatment is an issue of extreme importance for the healthcare community, and correct training for the identification and approach of suspected cases is essential. Our results allow us to conclude that the possible forms of presentation and associations are diverse. Body force consists of a highlighting mechanism for cases of child violence, deserving special attention for suspicion by professionals. Younger children, from 0 to 5 years old, especially those under 1 year old, are the target of greater vulnerability, and orthopedic fractures in this age group should be investigated actively and protectively. Serial X-rays and profile incidences can be interesting strategies for diagnostic optimization. Cranioencephalic and facial lesions demonstrate greater morbidity and mortality. Extreme ages (<1 year and between 13-18 years) may present suspicious age ranges due to higher prevalence in older adults and mortality in younger adults. Multicenter studies with greater sampling and uniformity of the data can provide additional information and be of great value for better understanding of the national scenario about the topic. We consider necessary educational strategies regarding medical assistance to child violence in medical schools and specialized training services as well as training teams in nursing services regarding the suspicion, identification, approach, management, conduct and follow-up of cases of ill-treatment in children and adolescents.

## References

[1] Weinstein SL, Flynn JM, Crawford HA (2021). Lovell and winter's pediatric orthopaedics.

[2] Estatuto da criança e do adolescente (1990). Lei número 8069. [Internet].

[3] Blangis F, Allali S, Cohen JF, Vabres N, Adamsbaum C, Rey-Salmon C (2021). Variations in Guidelines for Diagnosis of Child Physical Abuse in High-Income Countries: A Systematic Review. JAMA Netw Open.

[4] Ruaro AF, Meyer AT, Aguilar JAG, Hellu JJ, Custódio MD (1997). Síndrome da criança espancada. Rev Bras Ortop.

[5] Assis SG, Avanci JQ, Pesce RP, Ximenes LF (2009). The situation of Brazilian children and adolescents with regard to mental health and violence. Ciênc. saúde coletiva.

[6] Read J (2017). Do adult mental health services identify child abuse and neglect? A systematic review. Rev Inter Enf.

[7] Sink EL, Hyman JE, Matheny T, Georgopoulos G, Kleinman P (2011). Child abuse: the role of the orthopaedic surgeon in nonaccidental trauma. Clin Orthop Relat Res.

[8] Kemp AM, Dunstan F, Harrison S, Morris S, Mann M, Rolfe K (2008). Patterns of skeletal fractures in child abuse: systematic review. BMJ.

[9] Marine MB, Forbes-Amrhein MM (2021). Fractures of child abuse. Pediatr Radiol.

[10] Hornor G (2005). Physical abuse: Recognition and reporting. J Pediatr Health Care.

[11] Banaszkiewicz PA, Scotland TR, Myerscough EJ (2002). Fractures in children younger than age 1 year: importance of collaboration with child protection services. J Pediatr Orthop.

[12] Schilling S, Christian CW (2014). Child physical abuse and neglect. Child Adolesc Psychiatr Clin N Am.

[13] da Silva Franzin LC, Olandovski M, Vettorazzi ML, Werneck RI, Moysés SJ, Kusma SZ (2014). Child and adolescent abuse and neglect in the city of Curitiba, Brazil. Child Abuse Negl.

[14] Rosenfeld EH, Johnson B, Wesson DE, Shah SR, Vogel AM, Naik-Mathuria B (2020). Understanding non-accidental trauma in the United States: A national trauma databank study. J Pediatr Surg.

[15] Sheets LK, Leach ME, Koszewski IJ, Lessmeier AM, Nugent M, Simpson P (2013). Sentinel injuries in infants evaluated for child physical abuse. Pediatrics.

[16] Henry MK, Wood JN (2021). What's in a name? Sentinel injuries in abused infants. Pediatr Radiol.

[17] Nimkin K, Kleinman PK (2001). Imaging of child abuse. Radiol Clin North Am.

[18] Christian CW (2015). The Evaluation of Suspected Child Physical Abuse. Pediatrics.

[19] Karmazyn B, Duhn RD, Jennings SG, Wanner MR, Tahir B, Hibbard R (2012). Long bone fracture detection in suspected child abuse: contribution of lateral views. Pediatr Radiol.

[20] UNICEF, Fórum Brasileiro de Segurança Pública (FBSP) (2021). O Panorama da Violência Letal e Sexual contra Crianças e Adolescentes no Brasil. Comunicado de imprensa [Internet].

